# The Symptoms Caused by Intestinal Parasites

**Published:** 1905-01-28

**Authors:** 


### THE SYMPTOMS CAUSED BY INTESTINAL PARASITES.

The clinical conditions attributed to the presence of the several varieties of worms in the human intestine form a somewhat perplexing problem. On the one hand it is not uncommon to discover in the fæces conclusive evidence of the existence of animal parasites in the bowel, though the patient is entirely free from all symptoms of intestinal or other disturbance. On the other hand cases are constantly reported in which severe and even alarming symptoms have promptly ceased on the expulsion per rectum of one or more intestinal worms. Such symptoms have included those of local irritation in some instances, and in others, circulatory and nervous phenomena attributed to reflex disturbance. In a third group may be placed evidences of more or less extreme anæmia due either to abstraction of blood from the vessels of the alimentary canal or to the absorption of a poison produced by the parasite. These extreme contrasts can only be reconciled on the supposition that different individuals possess different degrees of susceptibility to the mechanical and toxic effects of the parasites. The publication of records of cases in which symptoms of various kinds have ceased after the expulsion of worms from the intestine has a useful and practical interest, inasmuch as it directs attention to a possible cause of numerous cases of obscure causation, and suggests that in such cases measures should be taken which will either confirm or exclude this suspicion. A systematic examination of the fæces or the use of a vermifuge has on more than one occasion announced a diagnosis which had previously been entirely unsuspected. A very striking case that may be quoted in support of this position is that of a male adult in whom prolonged constitutional disturbance, including a considerable degree of pyrexia, abruptly ceased when a round worm was vomited.[1] In another case,[2] the patient being a girl of 12 years, the temperature reached 104·3°; the other symptoms included headache, delirium, and some degree of abdominal distension so that the diagnosis seemed to rest between the cerebral form of typhoid fever and meningitis. On the fourth day of the illness, however, three doses of calomel were given and this was followed by the expulsion of a number of round worms and by the cessation of all the symptoms. The late Major A. E. Grant,[3] on the basis of a considerable Indian experience, stated that among the natives of that country the presence of ascarides was one of the commonest causes of fever and that many extraordinary and misleading symptoms were often produced by this parasite. He believed that the round worm was capable not only of wandering into the various passages which open into the alimentary canal but of actually penetrating the wall of the canal and so reaching other parts of the body. This latter proposition receives support from the statement that in occasional instances a round worm has been discovered in an abscess which has formed in the wall of the abdomen.[4] Another evil result attributed to a group of ascarides is the production of intestinal obstruction and a case of this order and attended with a fatal result has recently been recorded by Porot[5]; previous to this development there had been nothing in the history or symptoms of the case to suggest the presence of intestinal worms. In an analysis of the symptoms presented by 132 patients of ages from eight months to 33 years, Mr. Walter H. Haw[6] records a formidable list. Many of the symptoms included in this are more or less generally associated with a suspicion of the presence of intestinal worms. Others, such as jaundice, ascites, intestinal obstruction, vomiting, and fever hardly suggest such a diagnosis. It may be noted that among Mr. Haw's patients fever was noted in 41 cases (31 per cent.).

In connection with the presence of thread-worms in the colon, an interesting observation is recorded by Armand Ruffer.[7] In making a post-mortem examination on the body of a male Egyptian many oxyurides were observed in the large intestine, and also in the wall of the rectum three hard tumours, each about the size of a small nut. Smaller nodules were present in other parts of the colon. The mucous membrane over all these masses was quite free from congestion and ulceration, and the peritoneum in their neighbourhood showed no signs of inflammation. At first it was thought that the condition was due to the Bilharzia hæmatobia which is so common in the natives of Egypt. But further examination proved this to be a mistake. Each tumour was found to contain a calculus consisting of an amorphous yellow-brown substance with which were mixed numberless typical ova of the oxyuris vermicularis, in many of which were perfectly normal embryos. No traces of the adult worm were detected in any of the calculi. Evidently, as the surface of the mucous membrane was unbroken, the parent worm must have penetrated the intestinal wall and deposited its ova, which, acting as an irritant, produced localised thickenings, followed by calcification. The adult parasite, it may be presumed, either died and was absorbed, or found its way back again into the intestinal canal.

A more serious charge brought against intestinal worms is the possibility of producing appendicitis, or at least of symptoms which exactly simulate the ordinary form of appendicitis. Not only have these parasites been found in the appendix on operation but pain and other suggestions of appendicitis have disappeared on the effectual administration of a vermifuge. It would seem wise in any doubtful case carefully to examine the fæces for the presence of parasitic ova. Indeed there is wisdom in the recommendation[8] to do this in all cases of intestinal disease, just as it is the rule to examine the sputa in suspected disease of the bronchial tract and lungs.

[1] Brit. Med. Jour., July 15, 1893. [2] The Lancet, Sept. 26, 1903. [3] Ibid., Oct. 17, 1903. [4] Bain's Text-Book of Medical Practice (Longmans and Co., 1904). [5] Brit. Med. Jour. (Epitome, p. 93), Dec. 24, 1904. [6] Lancet, June 18, 1904. [7] Brit. Med. Jour., Jan. 26, 1901. [8] Lancet, March 23, 1901.

